# Nativity Status and Poly Tobacco Use among Young Adults in the United States

**DOI:** 10.3390/ijerph19031230

**Published:** 2022-01-22

**Authors:** Peace C. Okpala, Carrie Rosario, Melissa J. Dupont-Reyes, Michelle Y. Martin Romero, Md Towfiqul Alam, Hailey Paivanas, Sandra E. Echeverria

**Affiliations:** 1 Department of Public Health Education, University of North Carolina at Greensboro, Greensboro, NC 27412, USA; cmrosari@uncg.edu (C.R.); michellemartinromero@uncg.edu (M.Y.M.R.); m_alam@uncg.edu (M.T.A.); hnpaivan@uncg.edu (H.P.); seecheve@uncg.edu (S.E.E.); 2 Department of Epidemiology & Biostatistics, Texas A&M University, College Station, TX 77843, USA; melissadr@exchange.tamu.edu

**Keywords:** poly tobacco, nativity, young adults

## Abstract

**Introduction**: Young adults are the second largest segment of the immigrant population in the United States (US). Given recent trends in later age of initiation of tobacco use, we examined variation in use of tobacco products by nativity status for this population group. **Methods**: Our study included young adults 18–30 years of age sampled in the National Health Interview Survey (2015–2019), a nationally representative sample of the US population. We calculated prevalence of use of any and two or more tobacco products (cigarettes, cigars, pipes, e-cigarettes, and smokeless tobacco) for foreign-born (*n* = 3096) and US-born (*n* = 6811) young adults. Logistic regression models were adjusted for age, sex, race-ethnicity, education, and poverty, while accounting for the complex survey design. **Results**: Foreign-born young adults were significantly less likely to use any tobacco product (Cigarette = 7.3% vs. 10.7%; Cigar = 1.8% vs. 4.8%; E-cigarette = 2.3% vs. 4.5%, respectively; *p* < 0.01) or poly tobacco use (1.9% vs. 4.2%; *p* < 0.01) than US-born young adults. Adjusted regression models showed lower odds of poly tobacco use among the foreign-born than their US-born counterparts (Odds Ratio = 0.41, (95% Confidence Interval: 0.26–0.63)). **Conclusions**: The findings highlight the importance of targeted interventions by nativity status and further tobacco prevention efforts needed for the US-born.

## 1. Introduction

Despite the steady decline in the rate of tobacco use in the United States (US), it remains the leading cause of preventable deaths in the nation [1,2,3]. About 40 million US adults are current users and every day about 1600 youth initiate cigarette use [1]. In the US, about 500,000 people die each year due to tobacco use or exposure to secondhand smoke, and an estimated 16 million are currently diagnosed and live with a chronic illness, such as lung cancer, heart disease, stroke, and diabetes, caused by tobacco use [1]. Heart disease, which is associated with tobacco use, remains the leading cause of death in the US [1,4].

Surveillance data on tobacco use has shown that most adult users initiated tobacco consumption during their adolescent years, leading to the majority of prevention efforts focused on the adolescent population [5]. However, young adulthood is a critical age period where health behaviors are also adopted and a growing body of evidence suggests a later age of tobacco use initiation from adolescence to young adulthood [6,7]. Although there are varying definitions of the young adult period, several studies classify young adults as those 18–30 years of age [8,9]. Young adults are a particularly vulnerable target population of the tobacco industry because they are not completely covered by policies that limit the age of purchasing tobacco products and do not constitute a priority population for tobacco prevention efforts [10,11]. Additionally, young adults are an increasingly diverse segment of the US population. Currently, there are an estimated 44.8 million individuals who were born outside of the US, of which young adults less than 35 years of age represent 25.8% of the foreign-born. This age group has also been the second largest growing segment of the foreign-born population in the US over the past 30 years [12], but remains an understudied group in tobacco research.

Despite the growth and relevance of young adults, including the foreign-born, for tobacco research, little is known about tobacco product use in this age group, particularly regarding poly tobacco use. Poly tobacco product users have been shown to have a higher risk for nicotine addiction and dependence [13,14]. The odds for substance-use disorders are also two times higher for poly tobacco users compared to single tobacco product users [15]. Although prior research has shown that foreign born adults are less likely to consume tobacco products than their US born counterparts [4], we know little about patterns of poly tobacco use for young adults or whether tobacco use differs in the foreign-born [16]. In the present study, we examined patterns in poly tobacco use for young adults and determined differences by nativity status using a nationally representative sample of young adults. We hypothesized a significant difference in poly tobacco use between foreign-born and US-born young adults due to acculturation experiences and socioeconomic conditions that shape health differently in each of these groups, including the disruption of social networks and increased social isolation due to the migration process [17,18]. The study findings can inform planning of tobacco prevention and control for a fast-growing segment of the US population.

## 2. Materials and Methods

### 2.1. Data Source

The National Health and Interview Survey (NHIS) is a nationally representative cross-sectional study of the US population. Data from five years (2015–2019) were merged to increase the foreign-born sample. We limited our analysis to this study period because NHIS first began collecting data on various tobacco products in 2015. We do not include more recent waves of data (i.e., 2020) given disruptions in national surveillance systems caused by the COVID-19 pandemic [19]. NHIS participants self-report on a variety of health conditions during interviews conducted in their homes by professional interviewers. For purposes of this study, data from the NHIS adult, family, and person files were used.

### 2.2. Study Population

Our study population includes young adults aged 18–30 years of age following previous research with this age cut-off [8,9]. We further restricted the young adult population to the largest immigrant origin groups living in the US, which includes people of African, Asian, or Latin American origin. We excluded non-Latino Whites given the relatively small percentage of the population (<5%) who is foreign-born. For the study period from 2015 to 2019, NHIS sampled a total of 199,510 individuals. After restricting our study population to those 18–30 years of age and those who self-identify as Asian, Black, or Latino, our final analytic sample consisted of 9907 participants.

### 2.3. Study Variables

#### 2.3.1. Dependent Variable

We assessed consumption (yes/no) of each of the following 5 tobacco products: (1) cigarettes, (2) cigars, cigarillos, or filtered little cigars, (3) pipes, water pipes, or hookahs, (4) e-cigarettes, and (5) smokeless/chewable tobacco of interest. ‘Single’ tobacco use was defined as the use of only one tobacco product. ‘Any’ tobacco use was defined as the current use of any combination of tobacco products. ‘Poly’ tobacco use was defined as the use of 2 or more of the 5 tobacco products being studied. Participants were defined as current tobacco users if they reported consuming any of the 5 tobacco products in the last 30 days at the time of the survey.

#### 2.3.2. Independent Variable

Nativity status was based on participant self-report. The foreign-born population was defined as young adults who were not born in the United States or any of its territories. Foreign born young adults were compared to their US born counterparts (referent group). 

Confounders were selected *a priori* and include self-reported race/ethnicity (Asian, Black, and Latino origin), sex, age (18–30, 31–39, 40–49, 50–59, 60–69, 70 and above), education (less than high school graduate, high school graduate, some college, and college graduate), and poverty (below poverty threshold, above poverty threshold). We also adjusted for survey year to control for changes in tobacco consumption that may have occurred over the study period. Non-Latino Whites were excluded from analyses. The age variable was kept in continuous form given that the study population includes young adults 18–30 years of age. 

We also considered the role of social contexts in shaping tobacco consumption. Prior evidence has shown that poor housing quality and low neighborhood social cohesion, for example, are associated with increased tobacco use [20,21]. In NHIS, neighborhood social cohesion was measured with four questions assessing neighborhood trust, help, people to count on, and how close-knit residents feel about the neighborhood, using a 4-point Likert scale. We summed across the four measures to create a total neighborhood social cohesion score, with increasing score representing increasing neighborhood social cohesion. Although these measures were only available for 2015–2018 (not 2019), we ran exploratory models adjusting for neighborhood social cohesion to determine potential changes to our study results. 

### 2.4. Statistical Analyses

We merged 5 years of NHIS data (2015–2019), and all analyses included sampling weights to account for the complex sampling design and multiple years of study data. We generated descriptive statistics for the demographic characteristics of interest by tobacco use. Weighted prevalence estimates were conducted, and the Pearson chi square test was utilized to determine if there were significant associations between nativity groups. We fit binary logistic regression models to examine the association between nativity and poly tobacco use and use of cigarette, cigars, and e-cigarettes. Smokeless tobacco and pipe use were excluded from regression models given the small sample sizes. Model 1 represents Odds Ratio (OR) estimates for the crude association between nativity and tobacco use. Model 2 represents the fully adjusted model and includes age, sex, race/ethnicity, education, and poverty. All analyses were conducted using SAS software v. 9.4 with a 2-sided test of significance.

## 3. Results

Table 1 shows the weighted prevalence estimates of demographic characteristics by poly, any, and single tobacco use. The prevalence of poly, any and single tobacco use was significantly lower among foreign-born young adults (1.9%, 11.6%, 9.6%) compared to their US-born counterparts (4.2%, 17.6%, 13.3%), respectively. As educational attainment increased from less than high school degree to having a college degree or more, prevalence for poly, any, and single tobacco use decreased (*p* < 0.001). Those with less than a high school degree had the highest prevalence of poly tobacco use compared to those that had a high school degree, some college, or a college degree (4.4%, 3.8%, 2.7% and 2.1%), respectively. The rate is much higher for any tobacco use and those with less than a high school degree still represents the highest prevalence category, with about 20.8%, as compared to 16.8%, 15.1%, and 11.7% among those with a high school degree, some college, or a college degree, respectively. A similar pattern was also observed for single tobacco product use. Men had a significantly higher prevalence for poly tobacco use compared to women (5.5% vs. 1.6%), respectively (*p* < 0.001). The prevalence estimate for men who use only one tobacco product is 15.4% whereas for women it stands at 8.9%. Overall, both men and women had more single tobacco product users as compared to poly tobacco users. For race and ethnicity categories, non-Latino Black young adults had a significantly higher prevalence for poly tobacco use than Latinos or non-Latino Asians. Non-Latino Black young adults had a higher prevalence of poly tobacco use compared to Latinos and non-Latino Asians (4.9%, 2.9%, 2.8%), respectively. Non-Latino Whites were excluded from the sample due to a small percentage of foreign-born non-Latino Whites. For any tobacco use, non-Latino Black young adults also had the highest prevalence estimate compared to Latino and non-Latino Asians (19.6%, 14.1%, 12.7%), respectively. We also see a similar pattern of higher prevalence among non-Latino Black young adults for single tobacco product use when compared to Latinos and non-Latino Asians (14.6%, 11.2%, 9.9%), respectively. Participants below the poverty threshold had a higher prevalence for poly, any, and single tobacco use (4.1% vs. 3.6%, 16.7% vs. 3.6%, 12.5% vs. 11.9%), respectively, although differences were not statistically significant when compared to those above the poverty threshold.

Table 2 shows the weighted prevalence estimates of use of any of the five tobacco products by nativity status. Cigarettes were the most used tobacco product for both foreign-born and US-born young adults. This was followed by cigars, e-cigarettes, pipes, and smokeless tobacco. The US-born young adults had a slightly higher prevalence for cigar use (4.8%) compared to e-cigarette use (4.5%), this estimate excludes non-Latino Whites due to a small foreign-born sample size. Additionally, the US-born were significantly more likely to consume cigarettes (10.70% vs. 7.30%), cigars (4.8% vs. 1.8%), and e-cigarettes (4.5% vs. 2.3%), than the foreign-born (*p* < 0.001). There were no statistically significant differences by nativity status for smokeless tobacco and pipes.

Table 3 shows results from the logistic regression model. In Model 1 (crude estimate), foreign-born young adults have 55% lower odds for poly tobacco use compared to US-born young adults (Odds Ratio [OR]: 0.45, 95% Confidence Interval [CI]: 0.31–0.64). 05). In Model 2, after adjusting for age, sex, race/ethnicity, education, and poverty, this association remains, with foreign-born young adults having 59% lower odds for poly tobacco use compared to US-born young adults (OR = 0.41, 95% CI: 0.26–0.63, *p*-value <0.05). For any tobacco use, foreign-born young adults had 47% percent lower odds (OR = 0.53, 95% CI: 0.42–0.65), whereas for single tobacco use, they had 39% lower odds compared to their US-born counterparts (OR = 0.61, 95% CI: 0.49–0.79). We also fitted logistics regression models for each type of tobacco product (results were not included), and there was the same pattern of lower odds for tobacco use among foreign-born young adults compared to the US-born young adults. Models adjusted for neighborhood social cohesion for the period 2015–2018 showed no substantive changes in the association between nativity and tobacco use (data not shown).

## 4. Discussion

Our study provides new evidence on nativity differences in various measures of tobacco use for the young adult population living in the US. Results showed that for each of the five tobacco products examined, foreign-born young adults were significantly less likely to consume any of the tobacco products examined than their US-born counterparts. Cigarettes were the most consumed tobacco product for both foreign and US-born young adults. Moreover, poly, single, and any tobacco use was significantly lower among foreign born young adults compared to the US-born. There were no statistically significant differences by nativity status for pipe and smokeless tobacco. 

Findings from our study have important implications for tobacco policy adoption and prevention initiatives for young adults. Results corroborate prior research showing that cigarettes are the most commonly used tobacco product, although most studies have focused on the adult population [14]. Prior studies also indicate a lower prevalence of any tobacco use among the foreign-born population compared to the US-born [4,9,22], but few studies have examined differences in young adults. Results from our study indicated a consistent pattern of lower use of any, single, and poly tobacco product for foreign-born young adults compared to their US-born counterparts, ranging as high as a 6% difference for any tobacco use. These consistent differences in tobacco consumption suggest a critical role for policies to prevent tobacco use among the young adult population, especially the US-born. A study by Ickes et al. [23], for example, found that students who attend campuses without a comprehensive smoke-free policy were more like to report tobacco use on campus compared to students on a tobacco-free campus (65% vs. 36%), respectively. Our findings support the need for comprehensive smoke-free policies targeted at young adults.

Poly tobacco use can be influenced by several marketing techniques. Use of poly tobacco products has increased in recent years given the novelty of some of the products and marketing directed at young adults [24,25,26]. A study by Herrera et al. [24], found that increased exposure to free samples and marketing from the tobacco industry at night clubs/bars increased the number of products used among non-tobacco users 6 months later. Another study found that advertising of any tobacco product on Reddit (social media site) increased the odds for e-cigarette use among young adults [25,27]. These studies suggest young adults are a key population targeted in tobacco marketing. Additionally, contrary to prior research showing that about 6.9% of study participants were dual users of cigarettes and other tobacco products [28], we found that about 3.6% of our study population used two or more products. Although the previous study [28] included the general adult population in their sample, we advanced research by focusing on poly tobacco use patterns among young adults 18–30 years of age. 

Another factor that may influence tobacco use is neighborhood context and immigration-related factors. As previously noted, prior evidence shows that neighborhoods with substandard housing qualities increase tobacco use among residents [21] and low neighborhood cohesion is associated with smoking. When we adjusted for neighborhood social cohesion, our results remained substantively the same (data not shown). However, data were missing for 2019 and we were not able to adjust for other measures of the neighborhood context such as violence, tobacco product marketing, and smoking norms that may shape tobacco consumption. Additionally, similar to prior research, we found that the foreign-born have lower prevalence of single product use compared to the US-born population [1,29,30]. However, our study is unique in focusing on the young adult population and being able to compare single use and multiple tobacco products. One potential explanation for the lower single tobacco use is that foreign-born young adults may be from a lower socioeconomic status than the US-born, and thus do not have the purchasing power to consume tobacco products. Similarly, some research has shown that foreign-born healthier individuals with higher socio-economic status tend to stay in their home countries [31]. A second potential explanation is that some studies have found that parents’ health behaviors and higher parental monitoring of children may be responsible for lower prevalence of tobacco product use in foreign-born adolescents and young adults [31,32]. 

The findings from our study are subject to some limitations. Although NHIS is a nationally representative of the U.S. population, we restricted our sample to the young adult population representing the largest foreign-born groups in the U.S. Results may not generalize to other young adult groups such as non-Latino Whites. Self-reported data are also prone to recall bias. As a cross-sectional study, causal inference is limited because we are not able to demonstrate that nativity status led to the tobacco use patterns we observed. However, reverse causality is unlikely because tobacco use *per se* does not influence nativity status. Another limitation is sample size. Although the total foreign-born population sample was sufficient to detect statistically significant associations, the use of certain tobacco products had low numbers for some groups, especially non-Latino Black young adults, possibly limiting study power. 

## 5. Conclusions

This study provides several important implications for public health decision making, policy, and practice. The nativity differences in tobacco use are crucial to help guide tobacco prevention and control efforts among the young adult population. Findings from the study also suggest the need for further research on the protective factors of tobacco use among foreign-born young adults. This study contributes to the growing evidence on the need to prevent smoking initiation by expanding adolescent tobacco prevention programs to include the young adult population, as not all populations initiate tobacco use during adolescence. Our study supports expansion of tobacco prevention funding for young adults in order to ensure tobacco-free generations. Finally, findings suggest a need for the implementation of comprehensive tobacco-control policies and anti-tobacco campaigns especially targeted at the young adult population. Policies can also be enacted to limit the presence of tobacco stores in close proximity to college campuses. 

## Figures and Tables

**Table 1:** Weighted prevalence estimates for demographic characteristics by any, poly, and single tobacco use—National Health Interview Survey (2015–2019), *n* = 9907.

Demographic Characteristics	Poly Tobacco Use *n* (%)	Any Tobacco Use *n* (%)	Single Tobacco Use *n* (%)
**Nativity**			
Foreign Born	65 (1.9) **	343 (11.6) **	278 (9.6) **
U.S. Born	296 (4.2) **	1281 (17.6) **	985 (13.3) **
**Educational Attainment**			
Less than Highschool Grad	58 (4.4) *	280 (20.8) **	222 (16.3) **
Highschool Grad	121 (3.8) *	501 (16.8) **	380 (12.9) **
Some College	134 (3.7) *	580 (15.1) **	446 (11.4) **
College +	48 (2.1) *	263 (11.7) **	215 (9.5) **
**Sex**			
Male	259 (5.5) **	989 (20.9) **	730 (15.4) **
Female	103 (1.6) **	636 (10.5) **	533 (8.9) **
**Race/Ethnicity**			
Latino	159 (2.9) **	757 (14.1) **	598 (11.2) **
Non-Latino Black	149 (4.9) **	644 (19.6) **	495 (14.6) **
Non-Latino Asian	54 (2.8) **	224 (12.7) **	170 (9.9) **
**Income**			
Above Poverty Threshold	196 (3.6)	859 (3.6)	663 (11.9)
Below Poverty Threshold	89 (4.1)	400 (16.7)	311 (12.5)

Note. **: *p* < 0.001 *: *p* < 0.05; +: college or a more advanced degree.

**Table 2:** Prevalence estimate of 5 tobacco products’ use by nativity status among young adults age 18–30, NHIS (2015–2019), *n* = 9907.

Tobacco Product Type	Foreign Born *n* (%)	US Born *n* (%)
Cigarette	234 (7.3)	817 (10.7)
Cigar	54 (2.0)	323 (4.8)
E-Cigarettes	63 (2.3)	288 (4.5)
Pipes	63 (1.9)	168 (2.3)
Smokeless Tobacco	9 (0.5)	50 (0.5)

Note: Estimates were not statistically significant for pipes and smokeless tobacco.

**Table 3:** Logistic regression results for tobacco product use by nativity status, NHIS 2015–2019, (*n* = 9907).

	Poly Tobacco Use		Any Tobacco Use		Single Tobacco Use	
	Model 1 ^a^ UOR (95% CI)	Model 2 ^b^ AOR (95% CI)	Model 1 ^a^ UOR (95% CI)	Model 2 ^b^ AOR (95% CI)	Model 1 ^a^ UOR (95% CI)	Model 2 ^b^ AOR (95% CI)
Nativity						
US Born	**1**	**1**	**1**	**1**	**1**	**1**
Foreign Born	**0.45(0.31–0.64)**	**0.41(0.26–0.63)**	**0.61(0.52–0.72)**	**0.53(0.43–0.65)**	**0.69(0.58–0.82)**	**0.61(0.49–0.79)**

Note: bold indicates *p* < 0.05; ^a^: unadjusted model; ^b^: adjusted for age, sex, race, education, and income.

## Data Availability

The National Health Interview Survey (NHIS) data was used for the analyses. We extracted a merged data set from 2015 to 2019 from: https://nhis.ipums.org/nhis/ (accessed on 23 March 2021). The data is publicly available for research purposes.
